# The Impact of and Interaction between Diabetes and Frailty on Psychosocial Wellbeing and Mortality in Ireland

**DOI:** 10.3390/ijerph17249535

**Published:** 2020-12-19

**Authors:** Mark O’Donovan, Duygu Sezgin, Rónán O’Caoimh, Aaron Liew

**Affiliations:** 1 HRB Clinical Research Facility Cork, Mercy University Hospital, T12 WE28 Cork, Ireland; markodonovan@ucc.ie; 2 College of Medicine, Nursing and Health Sciences, National University of Ireland Galway, H91 TK33 Galway, Ireland; duygu.sezgin@nuigalway.ie; 3 Department of Geriatric Medicine, Mercy University Hospital, T12 WE28 Cork, Ireland; rocaoimh@muh.ie; 4 Department of Endocrinology, Portiuncula University Hospital, H53 T971 Galway, Ireland

**Keywords:** depression, diabetes, frailty, quality of life, mortality, self-rated health

## Abstract

Frailty in middle-aged and older adults is associated with diabetes-related complications. The impact of and interaction between diabetes and frailty on psychosocial wellbeing and mortality in Ireland for adults aged ≥50 years were assessed using data from the Survey of Health, Ageing and Retirement in Europe. Measures included diabetes status (self-reported), frailty phenotype (≥3/5 criteria), low self-rated health (“fair” or “poor”), depression screening (EURO-D index score ≥4), and low quality of life (QoL) (CASP-12 index score < 35). Among the 970 participants, those with diabetes (*n* = 87) were more likely to be frail (23% vs. 8%; *p* < 0.001), have low self-rated health (46% vs. 19%; *p* < 0.001), depression (25% vs. 17%; *p* = 0.070), and low QoL (25% vs. 18%, *p* = 0.085). Adjusting for diabetes, age and sex, frailty independently predicted low self-rated health (OR: 9.79 (5.85–16.36)), depression (9.82 (5.93–16.25)), and low QoL (8.52 (5.19–13.97)). Adjusting for frailty, age and sex, diabetes independently predicted low self-rated health (2.70 (1.63–4.47)). The age-sex adjusted mortality hazard ratio was highest for frailty with diabetes (4.67 (1.08–20.15)), followed by frailty without diabetes (2.86 (1.17–6.99)) and being non-frail with diabetes (1.76 (0.59–5.22)). Frailty independently predicts lower self-reported wellbeing and is associated with reduced survival, underpinning its role as an integral part of holistic diabetes care.

## 1. Introduction

Diabetes is prevalent and is increasing worldwide parallel with global ageing and the worsening obesity pandemic [1,2,3]. Optimal management aims to prevent microvascular and macrovascular complications as well as related consequences including functional decline, institutionalisation and death [4]. In Ireland, the self-reported prevalence of diabetes in 2007 was estimated to range from <1% in people aged 18–39 years to as high as 7% in women and 11% in men aged ≥70 years [2]. The prevalence of undiagnosed diabetes is also expected to be high for Irish older adults with an estimate of 1.8% for those aged 45 to 75 years [1]. More recently, the Global Burden of Disease Study (2019) estimated a higher prevalence for diabetes in those aged ≥70 years in Ireland at 24% (21–27%). Internationally, the highest prevalence estimates were Qatar (78%), Bahrain (74%), Fiji (64%), American Samoa (63%) and Marshall Islands (60%); and the highest prevalence estimates in Europe were for Czech Republic (43%), North Macedonia (37%), Cyprus (37%), Bosnia and Herzegovina (35%), Luxemburg (35%) [3].

The European Depression in Diabetes Research Consortium campaigned for an increased awareness and screening for psychosocial factors associated with diabetes such as depression and self-rated quality of life (QoL) as they are significant and important correlates of diabetes [5]. In Ireland, studies using the Hospital Anxiety and Depression Scale found that having diabetes is associated a higher prevalence of depressive symptoms (score ≥11/21) [6] and a significantly higher prevalence of severe depressive symptoms (score ≥15/21) [7]. Low QOL has also been observed for Irish diabetes patients aged 20–75 years according to Audit of Diabetes-Dependent Quality of Life scores [8]. The association between diabetes and depression is mediated by several factors which may include physical inactivity, poor self-management, diagnosis-related distress, and frailty in advanced diabetes [9]. 

Frailty is a multidimensional age-related syndrome; and while little consensus has been reached on an overall definition [10] some agreement has been reached on physical indicators of frailty [11]. While numerous tools exist, the most common are those measuring the frailty phenotype [12]. This approach usually characterises frailty as at least three of the following symptoms: wasting, muscle weakness, mobility, exhaustion, and low physical activity [13,14]. Recent studies have hypothesised that the endocrine system is particularly important in regards to frailty onset [15] and there is strong observational evidence for the association between frailty and diabetes [16]. While frailty in the general population has a strong association with depression [17,18], the overall impact of frailty on psychosocial wellbeing among people with diabetes has limited research with a small number of heterogeneous studies. 

The largest study to date, including 717 Australian men aged 70–89 years with diabetes, suggests that physical frailty (FRAIL scale) mediated approximately 15% of the association between diabetes duration and depression according to the Geriatric Depression Scale [19]. In contrast, another study including 288 patients with diabetes aged ≥65 years from Japan found no significant association between frailty (as measured by comprehensive geriatric assessment) and depression according to the Geriatric Depression Scale (*p* ≥ 0.05) but a significant association for QOL according to the questionnaires of Morale scale of the Philadelphia Geriatric Center (*p* < 0.05) [20]. A Chinese study including 637 patients aged ≥65 years with both diabetes and hypertension found that 42% of the patients were frail (Tilburg Frailty Indicator Scale) and that depression (CES-D-10) had a direct positive association with frailty [β = 0.326, 95% CI: 0.229–0.411] [21]. Another study from Canada including 41 participants aged 41 to 83 years with both diabetes and chronic kidney disease found frail participants (Edmonton Frail Scale) had significantly more depression (Major Depression Inventory, *p* = 0.005) and lower QoL scores (Short Form Health Survey-36, *p* ≤ 0.05) [22]. To date, no studies have assessed this in the Irish context. In addition to psychosocial wellbeing, frailty predicts mortality in the general population [23] as well as amongst those with diabetes [24,25]. 

Given the high and growing prevalence of diabetes in Ireland, and the association between diabetes and frailty with negative psychosocial factors such as depression and reduced QoL, we aimed to examine these associations in an Irish population sample. 

## 2. Materials and Methods 

**Figure 1:**
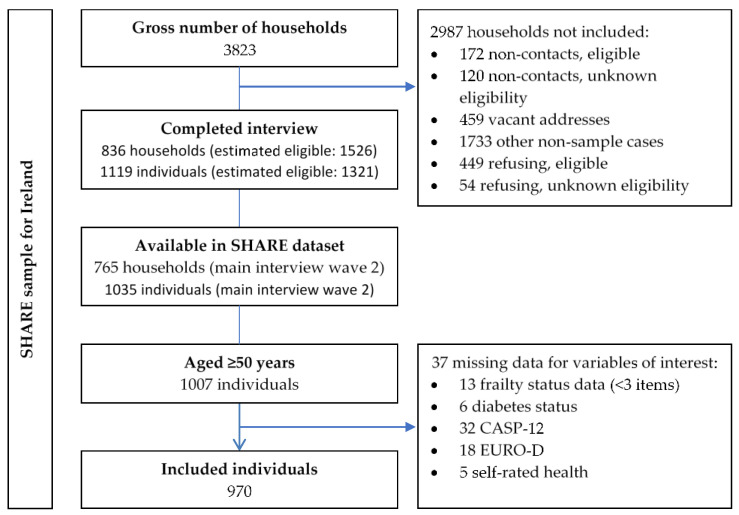
The SHARE sampling frame for Ireland with response rates and data availability [26,29].

This cross-sectional study is a secondary analysis of data from wave two [26] of the Survey of Health, Ageing and Retirement in Europe (SHARE) for Ireland. It also includes longitudinal mortality data assessed at wave three [27]. The SHARE sampling frame was taken from the civil register of Ireland with the sampling unit being individual addresses including nursing homes [28]. This was conducted with the support of the Economic and Social Research Institute with main interviews conducted between February and December 2007 by 105 trained interviewers [29]. In total, computer assisted personal interviews were completed face to face with over 1000 participants. Further details of the methodology of the SHARE have been published elsewhere [30]. Details on the SHARE sample for Ireland including response rates are presented in Figure 1.

For this secondary analysis, diabetes status was self-reported and determined from a positive response to either “Has a doctor ever told you that you have diabetes?” or “Do you currently take drugs at least once a week for diabetes?” QoL was measured using an abridged 12-item version of the Control, Autonomy, Self-realisation and Pleasure Scale (CASP-12), and a cut-off for low QoL of <35 [31]. Depressive symptoms were assessed using the 12-item EURO-D scale and a cut-off of ≥4 [32,33]. Self-rated health was assessed using a five-category question (poor, fair, good, very good, excellent) with low self-rated health (SRH) being defined as a response of either “poor” or “fair”.

Frailty was measured using a modified Fried Frailty Phenotype [34]. In brief, this modification uses measures of reduced appetite/food intake, exhaustion, weakness, walking difficulties, and low physical activity to approximate the five Fried criteria (weight-loss, exhaustion, weakness, slow walking and low physical activity [13]). Positive responses were added together with a score ≥3 classified as “frail” [34]. Participants were excluded if they were missing three or more values, as previously described [13]. 

Participants’ ages at the SHARE wave 2 (baseline of this study) were calculated from their month/year of birth and their month/year of interview. Post primary education status was determined from the SHARE education question taking a positive response as the International Standard Classification of Education (ISCED-97) codes 2–6, a negative response as ISCED-97 code 1 or “none”, and “still in school” or “other” as missing. The SHARE employment question was dichotomised into employed (“Employed or self-employed (including working for family business)”) and not employed (“unemployed”, “permanently sick or disabled”, “homemaker”, and “other”). Alcohol consumption was dichotomised using the question: “During the last 3 months, how often have you drunk any alcoholic beverages, like beer, cider, wine, spirits or cocktails?” taking a positive response as: “Almost every day” or “Five or six days a week” or “Three or four days a week” or “Once or twice a week” or “Once or twice a month” or “Less than once a month”; and a negative response as: “Not at all in the last 3 months”. Smoking status was determined from positive answers to both of the following questions: “Have you ever smoked cigarettes, cigars, cigarillos or a pipe daily for a period of at least one year?” and “Do you smoke at the present time?” 

Polypharmacy was defined as ≥5 drug types calculated from the sum of the positive responses to the SHARE question “Do you currently take drugs at least once a week for”: high blood cholesterol; high blood pressure; coronary or cerebrovascular diseases; other heart diseases; asthma; diabetes; joint pain or joint inflammation; other pain; sleep problems; anxiety or depression; osteoporosis (hormonal); osteoporosis (other); stomach burns; or chronic bronchitis. For comparability with later waves of the SHARE both osteoporosis (hormonal) and osteoporosis (other) were combined leaving a maximum total of 13 drugs. 

Diseases were self-reported from a question asking if a “doctor has told you that you have this condition, and that you are either currently being treated for or bothered by this condition”. These conditions included: hypercholesterolaemia, hypertension, heart attack (including myocardial infarction or coronary thrombosis or any other heart problem including congestive heart failure), stroke (or cerebral vascular disease), metastatic cancer (malignant tumour, including leukaemia or lymphoma, but excluding minor skin cancers), chronic lung disease (such as chronic bronchitis or emphysema), asthma, arthritis (including osteoarthritis, or rheumatism), osteoporosis, stomach/duodenal ulcer, Parkinson’s disease, cataracts, fractures (combined: “Hip fracture or femoral fracture” and “Other fractures”), and dementia (Alzheimer’s disease, dementia, organic brain syndrome, senility or any other serious memory impairment).

Cognitive impairment was measured based on previously described criteria [35], where questions on mathematical performance (five questions on percentages), verbal fluency (animal naming), and delayed recall were used, and cognitive impairment was defined as having all three of the following: an incorrect maths question, naming less than 15 animals and recalling less than three words. The health limiting activities question: “For the past six months at least, to what extent have you been limited because of a health problem in activities people usually do?” was scored as either “yes” (“Severely limited” or “Limited, but not severely”) or “no” (“Not limited”). 

The number of basic activities of daily living (BADL) limitations were calculated out of five using a summation of the positive responses for difficulties in the following: “bathing or showering”, “dressing, including shoes and socks”, “eating, cutting up food”, “using the toilet, including getting up or down”, and walking/transitioning (“walking 100 metres” or “getting up from chair” or “climbing several flights of stairs” or “climbing one flight of stairs” or “stooping, kneeling, crouching” or “walking across a room” or “getting in or out of bed”). The number of instrumental activities of daily living (IADL) limitations were calculated from a summation of positive responses for difficulties in the following seven items: “telephone calls”, “using a map in a strange place”, “preparing a hot meal”, “shopping for groceries”, “taking medications”, “doing work around the house or garden”, and “managing money”. 

The number of doctor visits was obtained from the question: “During the last twelve months, about how many times in total have you seen or talked to a medical doctor about your health? (Please exclude dentist visits and hospital stays, but include emergency room or outpatient clinic visits)”. Over-night hospital stay was from “During the last twelve months, have you been in a hospital overnight? (Please consider stays in medical, surgical, psychiatric or in any other specialized wards)”. The number nights in hospital was asked for those answering positively to the over-night hospital stay question as follows: “How often have you been a patient in a hospital overnight during the last twelve months?” Nursing home admission was asked as “During the last twelve months, have you been in a nursing home overnight?” which had three potential responses: “Yes, temporarily”, “Yes, permanently” or “No”. 

Differences in the survival time of participants were assessed using follow-up data from the SHARE wave 3 [27]. Where participants were coded as alive and had a wave 3 interview date, their follow-up duration was calculated as the wave 3 interview date (month/year) minus the wave 2 interview date (month/year). Where participants were coded as alive but did not have a wave 3 interview (or were missing the interview date), the date of the earliest wave 3 interview in Ireland was applied i.e., the minimum assumed follow-up. Where participants were coded as dead, their follow-up duration was calculated from their date of decease (month/year) minus their wave 2 interview date (month/year). 

Statistical analyses were conducted using IBM Statistics SPSS version 26. The continuous variables of interest (CASP-12 and EURO-D) were non-parametric according to Kolmogorov–Smirnov and Shapiro–Wilk tests, thus hypothesis tests used the non-parametric Mann–Whitney U Test. Hypothesis tests for categorical variables used the two-tailed Pearson’s chi-squared test. Pearson’s correlation and receiver operating characteristic (ROC) curve analysis were used to assess the correlation and predictive ability between the different assessments. Participants were categorised by diabetes and frailty status (frail with diabetes, frail without diabetes, non-frail with diabetes, non-frail without diabetes) and differences in mean survival duration were tested using Kaplan–Meier analysis. The significance of differences in survival durations were tested using the Mantel–Cox log-rank test. Survival hazard ratios (HR) adjusted for age and sex were calculated using Cox regression. All confidence intervals were calculated for a 95% significance level and hypothesis tests applied a statistically significant cut-off for *p*-values of ≤0.05.

## 3. Results

### 3.1. Sampling and Data Availability

From a total of 1035 Irish participants, 1007 were aged ≥50 years and 970 (96%) had sufficient data for frailty (≤2 missing items [13]) and no missing data for other variables of interest (diabetes status, CASP-12, 12 EURO-D items and self-rated health). Descriptive statistics of participants according to their diabetes and frailty status are presented in Table 1.

### 3.2. Baseline Differences According to Diabetes and Frailty

A total of 87 participants (9%) were classified as having self-reported diabetes and only 20 (2%) had both diabetes and frailty. Significant differences between frail and non-frail participants with diabetes were observed for alcohol consumption (20% vs. 61%, *p* = 0.001), polypharmacy (45% vs. 5%, *p* = 0.001), hypertension (70% vs. 45%, *p* = 0.048), stroke (25% vs. 6%, *p* = 0.014), arthritis (55% vs. 30%, *p* = 0.039), health limiting activities (95% vs. 51%, *p* < 0.001), median number of BADL limitations (2 vs. 1, *p* < 0.001), median number doctor visits (9 vs. 5, *p* = 0.022), and having an overnight hospital stay in the last year (40% vs. 15%, *p* = 0.015).

### 3.3. Differences in Frailty, Self-Rated Health, Depression, and QoL, According to Diabetes Status

**Figure 2:**
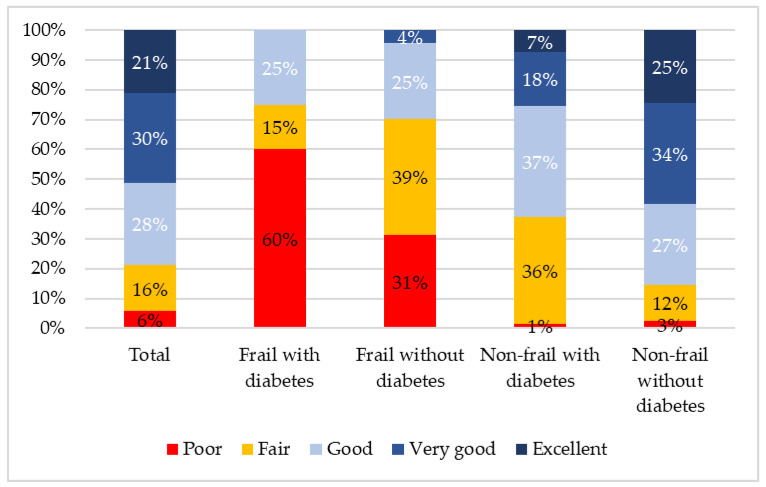
Differences in self-rated health according to diabetes and frailty status.

In this cohort, compared with those without diabetes, people with diabetes were more likely to be frail (23% vs. 8%; *p* < 0.001), have low SRH (46% vs. 19%; *p* < 0.001), depression (25% vs. 17%; *p* = 0.070) and low QoL (25% vs. 18%, *p* = 0.085). For participants with diabetes, 75% of those with frailty rated their health as low compared with 37% of non-frail participants (*p* = 0.003). For those without diabetes, the difference between frail and non-frail participants was 70% and 15% (*p* < 0.001). The five category differences in self-rated health are presented in Figure 2, illustrating that no one with frailty and diabetes rated their health as “excellent” or “very good” and the majority (60%) rated it as poor. These five category differences between participants who were frail and non-frail were highly statistically significant for both those with and without diabetes (*p* < 0.001). The difference between frail participants with and without diabetes was insignificant (*p* = 0.077).

Approximately 60% of frail participants screened positive for depression irrespective of diabetes status (*p* = 0.981). Being frail was significantly associated with higher depression scores for both those with diabetes (60% vs. 15%, *p* < 0.001) and without diabetes (60% vs. 14%, *p* < 0.001). Frailty was negatively associated with CASP-12 scores for both those with and without diabetes (*p* < 0.001). Results for the individual components of EURO-D and CASP-12 index are presented in Table 2. 

### 3.4. Overlap and Agreement between Measures

**Figure 3:**
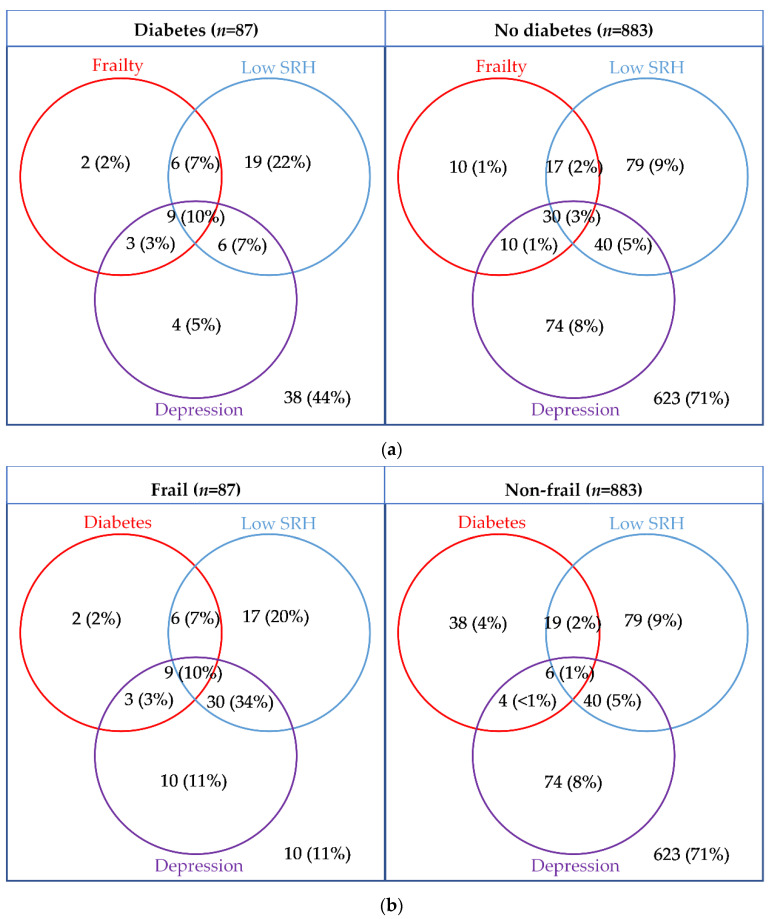
Venn diagrams presenting the overlap between diabetes or frailty, with depression (EURO-D ≥ 4), and low self-rated health (poor/fair) presented by (**a**) diabetes status and (**b**) frailty status.

The overlap between frailty, low SRH and depression are presented in Figure 3. Participants with diabetes were more likely to be frail, have low SRH and depression (10% vs. 3%; *p* = 0.002), and were more likely to have at least one of the three conditions (56% vs. 29%, *p* < 0.001).

**Figure 4:**
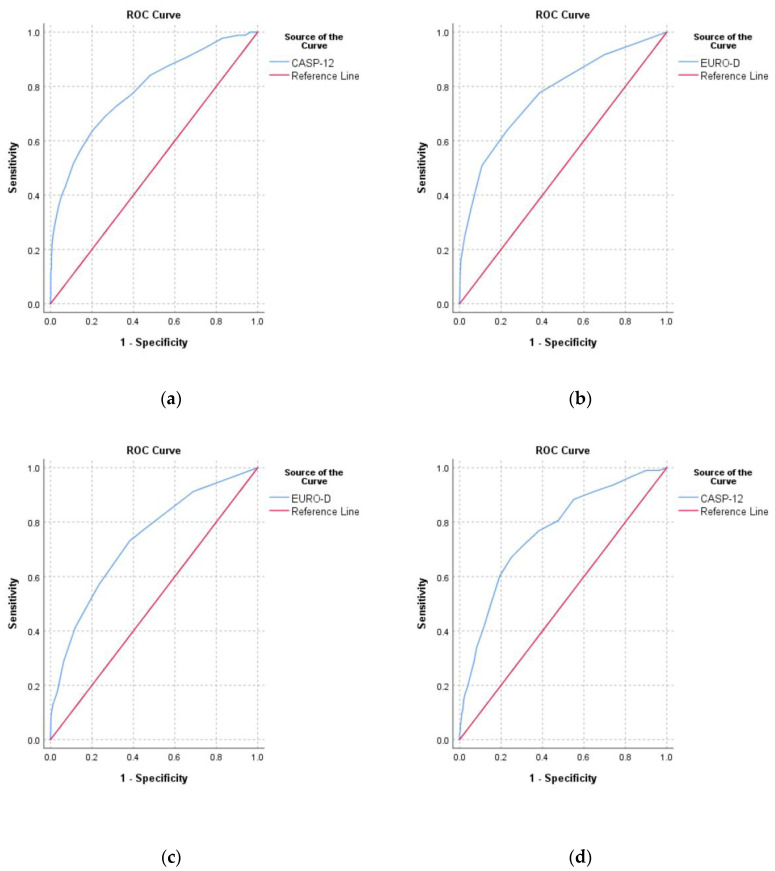
Receiver operating characteristic curves for (**a**) predicting depression (EURO-D ≥ 4) from the CASP-12 score; (**b**) predicting low quality of life (CASP-12 < 35) from the EURO-D score; (**c**) predicting low self-rated health from the EURO-D score, and (**d**) predicting low self-rated health from the CASP-12 score.

The correlation between CASP-12 score and EURO-D score was moderate (r = −0.53, *p* < 0.001). The area under the curve (AUC) for predicating depression from the CASP-12 score was 0.79 (0.75–0.83) and for predicting low QoL from EURO-D score it was 0.77 (0.73–0.81) (Figure 4). For predicting low SRH, the EURO-D score had an AUC of 0.73 (0.69–0.77) and the CASP-12 score had an AUC of 0.76 (0.73–0.80).

As illustrated in Table 3, diabetes when adjusted for age, sex, and frailty was significantly associated with low SRH (OR: 2.70 (1.63–4.47)) but had an insignificant association with depression, low QoL, or death. Frailty was a significant predictor of all four variables (low SRH, depression, low QoL, and death), adjusting for age, sex, and diabetes.

### 3.5. Survival Analysis

**Figure 5:**
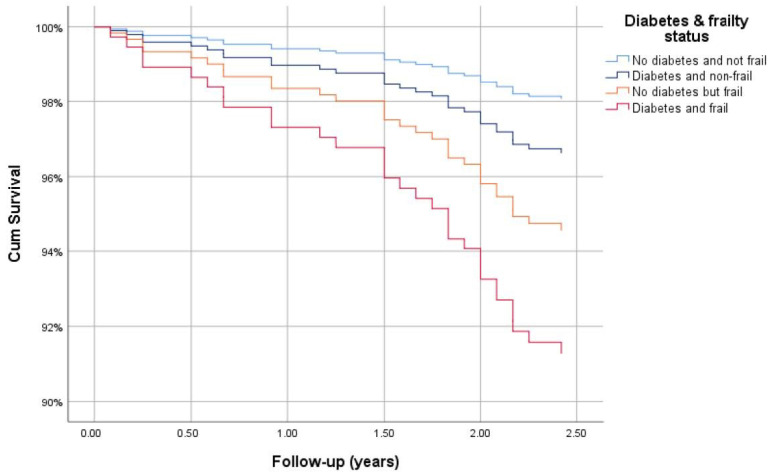
Cox regression survival curve for those with diabetes and frailty, adjusted for age and sex.

At wave 3, 808 out of 970 (83%) participants had a known survival status with a total of 46 deaths (Table 3). Of those who died, 32 had a date of decease available (month and year), thus a total of 794 participants had follow-up duration data. The interview date for wave 3 was missing for some participants (117/794) and for those the date of the earliest wave 3 interview was used. The number of deaths by diabetes and frailty status were as follows: non-frail without diabetes (18/669), non-frail with diabetes (4/55), frailty without diabetes (8/55), and frail with diabetes (2/15). The overall mean (SD) follow-up was 2.94 (0.53) years ranging from 0.08 to 3.92 years. According to Kaplan–Meier analysis, the shortest length of mean survival was 2.90 (95% CI: 2.66–3.14) years for those with frailty and diabetes, followed by those who were frail without diabetes 3.50 (3.26–3.73) years, non-frail with diabetes 3.59 (3.45–3.74) years and non-frail without diabetes 3.85 (3.81–3.88) years (*p* < 0.001). Similarly, after adjusting for age and sex, compared with those who were non-frail without diabetes, the survival hazard ratio was highest for those who were frail with diabetes 4.67 (1.08–20.15, *p* = 0.039), followed by those who were frail without diabetes 2.86 (1.17–6.99, *p* = 0.021) and finally those who were non-frail with diabetes 1.76 (0.59–5.22, *p* = 0.308) (Figure 5).

## 4. Discussion

This paper examines the relationship between diabetes, frailty, self-rated health, QoL, depression and mortality in middle aged and older Irish adults participating in the SHARE. Diabetes was associated with a significantly higher prevalence of frailty, and was an independent predictor of low SRH when adjusted for differences in age, sex and frailty status. Both depression (*p* = 0.07) and low QoL (*p* = 0.085) were more common for those with diabetes but did not reach statistical significance. Frailty was an independent predictor of low SRH, depression, low QoL, and death after adjusting for differences in age, sex, and diabetes status. Moreover, results from the survival analyses illustrated the lowest survival time was for those with both frailty and diabetes followed by frailty without diabetes, non-frail with diabetes and non-frail without diabetes, and these differences remained after adjusting for age and sex.

In Ireland, there have been conflicting results found on the association between diabetes and psychosocial factors. The CLARITY study [7] conducted in Ireland with 566 adults aged ≥50 found that multimorbidity but not type 2 diabetes was a statistically significant predictor of baseline depression. However, in another Irish study [6] with 1456 participants aged from 20 to 75 years, diabetic complications and uncertainty about glycaemic control were significant predictors of baseline depression (measured with Hospital Anxiety and Depression Scale). In The West of Ireland Diabetic Foot Study [36], including 563 people with diabetes, depression (measured with Hospital Anxiety and Depression Scale) was associated with a modified neuropathy disability score and symptoms including intermittent claudication, night pain, rest pain, and neuropathy symptom score. Overall, these findings suggest that pain and functional difficulties were the strongest correlates of depression in people with diabetes.

The association between diabetes and frailty was also expected with previous studies having hypothesised that the endocrine system is particularly important in regards to frailty onset [15]. An analysis of 493,737 UK Biobank participants [16] confirmed that frailty was a cross-sectionally associated with diabetes, and longitudinally associated with increased mortality, adjusting for key confounders. We further postulated that differences in frailty between those with and without diabetes may explain a large amount of the difference in psychosocial wellbeing between these groups which is supported by a previous study [19]. The association of frailty with depression and QoL is well illustrated in the general population [17,37] and this study found significant associations for both those with and without diabetes. 

In fact, when analysed based on frailty status, little difference was observed between participants with and without diabetes for either depression or QoL. This suggests that symptoms related to frailty are a stronger predictor of reduced wellbeing than the presence of diabetes. This also provides additional support for the need to reduce frailty in patients with diabetes, which has been highlighted in several recent international guidelines [38,39]. It has also been argued that the underlying causes of frailty may be different for people with diabetes [40,41,42] and further research is needed to assess if the mechanism of frailty is different in those with diabetes.

The association of both frailty and diabetes with lower self-rated health is another important finding with previous studies having demonstrated that self-rated health is an important predictor of mortality [43] and functional decline [44] in the general population. Specifically for those with diabetes, self-rated health has been shown to independently predict mortality after adjusting for key confounders [45]. In this cohort, low SHR was also found to be an independent predictor of depression and low QoL adjusting for age, sex, diabetes status, and frailty status. While not reaching statistical significance (*p* = 0.08), this study suggested that frail participants with diabetes rate their health considerably lower than those with frailty and no diabetes. Additional research is needed to understand if the health of those with diabetes and frailty is objectively worse than those with frailty and no diabetes.

This study has several limitations. Firstly, the sample size for those with diabetes and frailty was small (*n* = 20/970) which increases the likelihood of a Type 2 error and reduces the statistical power of the study. This further limited the ability to perform regression analysis and interpret the results reliably. Furthermore, there were multiple comparisons increasing the likelihood of multiplicity. In addition, while this study includes most available SHARE participants with 970 participants from 765 households, this only represents approximately 37% of the total estimated eligible sample for Ireland [29] making the chances of selection bias relatively high. The questions used in the SHARE may also have limited accuracy with diabetes status being self-reported. Similarly, depression was determined using a screening tool, the EURO-D scale, rather than a gold standard assessment. This said, a study from the USA suggests that self-reported diabetes has a 66.0% sensitivity and a 99.7% specificity for diabetes [46], and the EURO-D scale is estimated to have 86% sensitivity and 84% specificity for an ICD-10 depressive episode [33], suggesting that these may have sufficient accuracy. Regarding the mortality and survival analysis, there were missing data on survival status or date of death for 176 (18%) participants. There were also a further 117 (13%) individuals who were missing an interview date at wave 3 and were given an estimated follow-up time using the date of the earliest wave 3 interview.

## 5. Conclusions

Frailty was associated with a high proportion of depressive symptoms and lower QoL among Irish people with diabetes participating in the SHARE. Participants with diabetes were more likely to be frail, and the findings of this study suggest that differences in frailty between those with and without diabetes can explain the differences in psychosocial wellbeing. However, people with diabetes had lower self-rated health that was independent of frailty status. In relation to mortality, the lowest survival time was for those with both frailty and diabetes followed by frailty without diabetes, those who were non-frail with diabetes, and non-frail without diabetes. These differences in survival were also observed after adjusting for age and sex. Further research is needed to assess if frailty and diabetes are independent predictors of psychosocial wellbeing and mortality and whether the combination of frailty and diabetes is worse than frailty in the general population. This has important implications for the management of diabetes, particularly given the high incidence [47] and prevalence of frailty in Europe [48] and globally [49]. Frailty and its association with depression, reduced QoL and lower SRH should be considered as an integral part of holistic diabetes care. As well as implementing approaches to reduce frailty onset, psychosocial interventions should be developed to prevent and manage psychosocial consequences amongst those with diabetes with and without established frailty.

## Figures and Tables

**Table 1:** Characteristics of participants according to their diabetes and frailty status presented as the number (percentage) or median (inter quartile range).

Characteristics ^1^	Total *n* = 970	Diabetes and Frail *n* = 20	Diabetes and Non-Frail *n* = 67	Difference *p*-Value	No Diabetes but Frail *n* = 67	No Diabetes and not Frail *n* = 816	Difference *p*-Value
Age	62.71 (14.75)	67.50 (11.00)	69.00 (15.83)	0.268	70.75 (17.83)	61.58 (13.46)	<0.001
Sex (Female)	525 (54.1%)	13 (65.0%)	29 (43.3%)	0.088	44 (65.7%)	439 (53.8%)	0.061
BMI (kg/m^2^)	26.46 (6.63)	29.56 (5.66)	28.89 (7.42)	0.535	27.34 (9.37)	26.22 (6.32)	0.582
Education (post-primary)	708 (73.2%)	14 (70.0%)	44 (65.7%)	0.719	23 (34.3%)	627 (77.1%)	<0.001
Employed	344 (35.6%)	3 (15%)	13 (19.4%)	0.656	0 (0%)	328 (40.3%)	<0.001
Drink alcohol	657 (68%)	4 (20%)	41 (61.2%)	0.001	32 (47.8%)	580 (71.4%)	<0.001
Smoke	168 (33.3%)	3 (27.3%)	9 (23.1%)	0.774	18 (48.6%)	138 (33%)	0.055
Polypharmacy (≥5 drugs)	35 (3.6%)	9 (45%)	4 (6%)	<0.001	7 (10.6%)	15 (1.8%)	<0.001
Hypercholesterolaemia	268 (27.6%)	10 (50%)	21 (31.3%)	0.126	20 (29.9%)	217 (26.6%)	0.563
Hypertension	285 (29.4%)	14 (70%)	30 (44.8%)	0.048	29 (43.3%)	212 (26%)	0.002
Heart attack	72 (7.4%)	3 (15%)	8 (11.9%)	0.718	8 (11.9%)	53 (6.5%)	0.091
Stroke	37 (3.8%)	5 (25%)	4 (6%)	0.014	7 (10.4%)	21 (2.6%)	<0.001
Metastatic cancer	47 (4.8%)	1 (5%)	6 (9%)	0.568	7 (10.4%)	33 (4%)	0.015
Chronic lung disease	33 (3.4%)	2 (10%)	1 (1.5%)	0.067	8 (11.9%)	22 (2.7%)	<0.001
Asthma	74 (7.6%)	3 (15%)	6 (9%)	0.436	9 (13.4%)	56 (6.9%)	0.048
Arthritis	213 (22%)	11 (55%)	20 (29.9%)	0.039	39 (58.2%)	143 (17.5%)	<0.001
Osteoporosis	57 (5.9%)	1 (5%)	2 (3%)	0.665	11 (16.4%)	43 (5.3%)	<0.001
Stomach/duodenal ulcer	71 (7.3%)	3 (15%)	4 (6%)	0.193	8 (11.9%)	56 (6.9%)	0.123
Parkinson’s disease	6 (0.6%)	1 (5%)	0 (0%)	0.066	3 (4.5%)	2 (0.2%)	<0.001
Cataracts	48 (4.9%)	2 (10%)	8 (11.9%)	0.811	7 (10.4%)	31 (3.8%)	0.010
Fractures	59 (6.1%)	1 (5%)	6 (9%)	0.568	14 (20.9%)	38 (4.7%)	<0.001
Dementia	6 (0.6%)	0 (0%)	1 (1.5%)	0.583	1 (1.5%)	4 (0.5%)	0.293
Cognitive impairment	132 (13.7%)	9 (45%)	18 (27.3%)	0.135	21 (32.3%)	84 (10.4%)	<0.001
Health limits activities	298 (30.7%)	19 (95%)	34 (50.7%)	<0.001	61 (91%)	184 (22.5%)	<0.001
Median BADL limitations	0.00 (1.00)	2.00 (2.00)	1.00 (1.00)	<0.001	2.00 (2.00)	0.00 (1.00)	<0.001
Median IADL limitations	0.00 (0.00)	2.00 (3.00)	0.00 (0.00)	0.063	1.00 (2.00)	0.00 (0.00)	<0.001
Number doctor visits	3.00 (4.00)	9.00 (8.50)	5.00 (9.00)	0.022	6.00 (8.00)	2.00 (3.00)	<0.001
Over-night hospital stay	137 (14.1%)	8 (40%)	10 (14.9%)	0.015	26 (38.8%)	93 (11.4%)	<0.001
Number nights in hospital	7.00 (13.00)	5.50 (12.00)	6.50 (5.00)	0.924	14.00 (14.00)	5.00 (13.00)	0.004
Nursing home temporary	8 (0.8%)	0 (0%)	0 (0%)	0.358	4 (6%)	4 (0.5%)	<0.001
Nursing home permanent	8 (0.8%)	1 (5.0%)	1 (1.5%)	0.358	0 (0%)	6 (0.7%)	<0.001
Appetite/food intake	95 (9.8%)	12 (60.0%)	4 (6.0%)	<0.001	25 (37.3%)	54 (6.6%)	<0.001
Exhaustion	301 (31.0%)	16 (80.0%)	23 (34.3%)	<0.001	63 (94%)	199 (24.4%)	<0.001
Weakness	105 (13.6%)	8 (57.1%)	7 (12.3%)	<0.001	26 (65%)	64 (9.7%)	<0.001
Walking difficulties	131 (13.5%)	17 (85.0%)	9 (13.4%)	<0.001	58 (86.6%)	47 (5.8%)	<0.001
Low physical activity	143 (14.8%)	15 (75.0%)	10 (14.9%)	<0.001	54 (80.6%)	64 (7.9%)	<0.001

^1^ Note that some questions were not answered by all participants and percentages apply to those with available data.

**Table 2:** Differences between EURO-D and CASP-12 scores and their components for participants by diabetes and frailty status presented as the number (percentage) or median (inter quartile range).

Characteristics ^1^	Total *n* = 970	Diabetes and Frail *n* = 20	Diabetes and Non-Frail *n* = 67	Difference *p*-Value	No Diabetes but Frail *n* = 67	No Diabetes and Not Frail *n* = 816	Difference *p*-Value
Affective suffering	420 (43.3%)	12 (60%)	29 (43.3%)	0.189	49 (73.1%)	330 (40.4%)	<0.001
Motivation	214 (22.1%)	12 (60%)	13 (19.4%)	<0.001	42 (62.7%)	147 (18.0%)	<0.001
Frailty items	336 (34.6%)	18 (90%)	23 (34.3%)	<0.001	63 (94%)	232 (28.4%)	<0.001
Other EURO-D items	414 (42.7%)	13 (65%)	30 (44.8%)	0.112	42 (62.7%)	329 (40.3%)	<0.001
**Total EURO-D score**	176 (1.0%)	4.5 (5.5)	1.0 (2.0)	<0.001	4.0 (3.0)	1.0 (3.0)	<0.001
**Depressed (score ≥ 4)**	176 (21.5%)	12 (60.0%)	10 (14.9%)	<0.001	40 (59.7%)	114 (14.0%)	<0.001
Limitation control	9.00 (3.00)	6.50 (3.00)	8.00 (3.00)	0.023	6.00 (3.00)	9.00 (2.00)	<0.001
Limitation autonomy	9.00 (3.00)	9.00 (2.50)	10.00 (3.00)	0.101	9.00 (3.00)	9.00 (3.00)	0.005
Limitation self-realization	10.00 (3.00)	7.00 (4.50)	10.00 (2.00)	<0.001	7.00 (3.00)	10.00 (3.00)	<0.001
Limitation pleasure	12.00 (1.00)	10.00 (4.50)	12.00 (1.00)	0.007	11.00 (3.00)	12.00 (1.00)	<0.001
**Total CASP-12 score**	40.00 (7.00)	35.50 (11.00)	39.00 (6.00)	<0.001	32.00 (9.00)	40.00 (6.00)	<0.001
**Low QoL (CASP-12 < 35)**	179 (18.5%)	9 (45.0%)	13 (19.4%)	0.021	41 (61.2%)	116 (14.2%)	<0.001

^1^ Affective suffering includes: sadness/depression, pessimism, suicidality, and tearfulness; Motivation includes: reduced interest, poor concentration, and low enjoyment. Frailty items includes: reduced appetite and fatigue. Other EURO-D items includes: guilt, sleep problems, and irritability. Control includes: age prevents you from doing the things you would like to do? what happens to you is out of your control? feeling left out of things? Autonomy includes: you can do the things that you want to do? family responsibilities prevent you from doing what you want to do?; shortage of money stops you from doing the things you want to do?; Pleasure includes: look forward to each day?; feel that your life has meaning?; look back on your life with a sense of happiness? Self-realization includes: feel full of energy these days? feel that life is full of opportunities?; feel that the future looks good for you?

**Table 3:** Odds ratios (95% CI) for predicting low self-rated health, depression, and low quality of life and death using different models with diabetes, frailty, age, and sex.

Models	Variables	Low SRH ^1^ (*n* = 206/970)	Depression ^2^ (*n* = 176/970)	Low QoL ^3^ (*n* = 179/970)	Dead ^4^ (*n* = 46/808)
Model 1	Diabetes	3.68 (2.33–5.79)	1.60 (0.96–2.68)	1.57 (0.94–2.61)	2.65 (1.23–5.74)
Model 2	Frailty	12.73 (7.74–20.93)	9.09 (5.69–14.53)	7.90 (4.97–12.57)	4.13 (2.03–8.39)
Model 3	Diabetes	2.97 (1.80–4.91)	1.06 (0.59–1.89)	1.06 (0.60–1.88)	2.10 (0.94–4.69)
	Frailty	11.62 (7.01–19.24)	9.02 (5.60–14.52)	7.83 (4.88–12.55)	3.67 (1.77–7.59)
Model 4	Diabetes	2.70 (1.63–4.47)	1.14 (0.63–2.07)	1.07 (0.60–1.91)	1.81 (0.81–4.07)
	Frailty	9.79 (5.85–16.36)	9.82 (5.93–16.25)	8.52 (5.19–13.97)	2.69 (1.25–5.81)
	Age (years)	1.03 (1.01–1.05)	0.98 (0.96–1.00)	0.99 (0.97–1.01)	1.10 (1.07–1.14)
	Sex (female)	1.11 (0.79–1.56)	1.54 (1.07–2.20)	0.83 (0.59–1.17)	0.73 (0.39–1.37)
Model 5	Diabetes	2.76 (1.63–4.68)	0.99 (0.53–1.86)	0.85 (0.46–1.59)	1.67 (0.73–3.80)
	Frailty	4.81 (2.73–8.45)	3.90 (2.19–6.93)	3.01 (1.70–5.35)	1.50 (0.62–3.66)
	Low SR health	-	2.59 (1.68–4.01)	2.95 (1.93–4.51)	1.99 0.99–4.01)
	Depression	2.63 (1.70–4.08)	-	5.58 (3.71–8.39)	1.54 (0.66–3.60)
	Low QoL	2.90 (1.89–4.44)	5.59 (3.71–8.42)	-	1.25 (0.55–2.84)
	Age (years)	1.04 (1.02–1.06)	0.98 (0.96–1.00)	0.99 (0.97–1.01)	1.10 (1.07–1.14)
	Sex (female)	1.07 (0.75–1.54)	1.76 (1.19–2.61)	0.68 (0.46–0.99)	0.68 (0.36–1.30)

^1^ Low SRH= “poor” or “fair”; ^2^ Depression = EURO-D ≥ 4; ^3^ Low QoL = CASP-12 < 35; ^4^ Died = dead at the SHARE wave 3.
